# *In silico* characterisation of the complete Ly6 protein family in *Fasciola gigantica* supported through transcriptomics of the newly-excysted juveniles†

**DOI:** 10.1039/d1mo00254f

**Published:** 2021-11-08

**Authors:** Sarah D. Davey, Iain W. Chalmers, Narcis Fernandez-Fuentes, Martin T. Swain, Dan Smith, Syed M. Abbas Abidi, Mohammad K. Saifullah, Muthusamy Raman, Gopalakrishnan Ravikumar, Paul McVeigh, Aaron G. Maule, Peter M. Brophy, Russell M. Morphew

**Affiliations:** Institute of Biological, Environmental & Rural Sciences (IBERS) Aberystwyth Ceredigion UK iwc@aber.ac.uk; Computational and Analytical Sciences, Rothamsted Research Harpenden Hertfordshire UK; Department of Zoology, Aligarh Muslim University Aligarh Uttar Pradesh 202001 India; Tamil Nadu Veterinary and Animal Sciences University Chennai Tamil Nadu 600051 India; School of Biological Sciences, Queen's University Belfast Belfast UK

## Abstract

*Fasciola gigantica* is one of the aetiological trematodes associated with fascioliasis, which heavily impacts food-production systems and human and animal welfare on a global scale. In the absence of a vaccine, fascioliasis control and treatment is restricted to pasture management, such as clean grazing, and a limited array of chemotherapies, to which signs of resistance are beginning to appear. Research into novel control strategies is therefore urgently required and the advent of ‘omics technologies presents considerable opportunity for novel drug and vaccine target discovery. Here, interrogation of the first available *F. gigantica* newly excysted juvenile (NEJ) transcriptome revealed several protein families of current interest to parasitic flatworm vaccine research, including orthologues of mammalian complement regulator CD59 of the Ly6 family. Ly6 proteins have previously been identified on the tegument of *Schistosoma mansoni* and induced protective immunity in vaccination trials. Incorporating the recently available *F. gigantica* genome, the current work revealed 20 novel Ly6 family members in *F. gigantica* and, in parallel, significantly extended the *F. hepatica* complement from 3 to 18 members. Phylogenetic analysis revealed several distinct clades within the family, some of which are unique to *Fasciola spp.* trematodes. Analysis of available proteomic databases also revealed three of the newly discovered FhLy6s were present in extracellular vesicles, which have previously been prioritised in studying the host-parasite interface. The presentation of this new transcriptomic resource, in addition to the Ly6 family proteins here identified, represents a wealth of opportunity for future vaccine research.

## Abstract 2

Incorporating the recently available *F. gigantica* genome, the current work revealed 20 novel Ly6 family members in *F. gigantica* and, in parallel, significantly extended the *F. hepatica* complement from 3 to 18 members.

## Graphical abstract

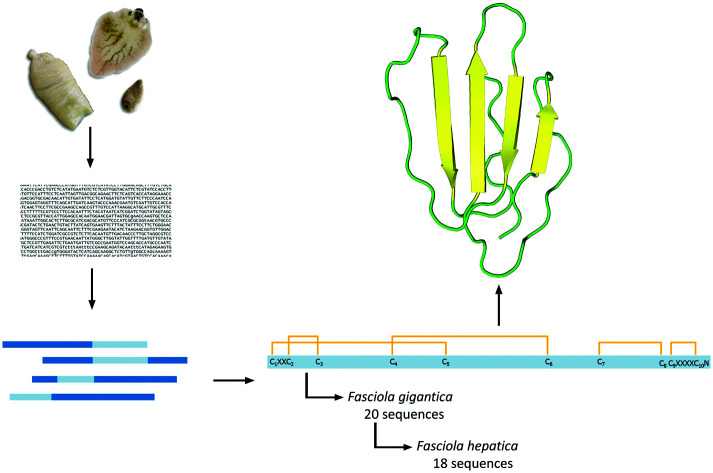

## Introduction

In recent decades, the advent of high-throughput sequencing and bioinformatics-based computational processing has allowed parasitologists to access a wide array of genetic data.^1^ Publication of this data has been vital in developing a better understanding of the molecular processes, key genes and proteins involved in parasitism, as well as clarifying evolutionary relationships between the species.^2,3^

An area of research that could benefit from the use of ‘omics data is the study of *Fasciola spp.* trematodes, which are aetiological agents in the zoonotic disease, fascioliasis.^4,5^*Fasciola spp.* trematodes have complex life cycles and undergo development in the environment, molluscan intermediate host and definitive mammalian hosts. The juvenile stages are particularly relevant to the pathology of this disease due to the migration of the parasite through the host abdominal cavity to the liver, resulting in significant mechanical and inflammatory damage to the host.^6^ Despite the global importance of this disease, relatively little genomic or bioinformatic data has been historically available for these parasites, thus there is substantial opportunity to expand on the understanding of fascioliasis’ molecular underpinning.^7^

*Fasciola gigantica* lacked complete genomic representation until very recently, with the independent publication of two new draft genomes.^8,9^ Consequently, research into *F. gigantica* molecular biology has been reliant on limited transcriptomic data. Whole-organism transcriptomes are currently available for the egg, miracidial, redial, cercarial, later juvenile (42 and 70 days post infection) and adult stages, in addition to smaller datasets which focus specifically on the host-parasite interface and pathogenicity.^10–14^ The newly excysted juvenile (NEJ) has been entirely neglected, with no transcriptomic datasets publicly available and most studies focusing on small subsets of data, such as specific genes for RNAi interference.^15^ Considering the importance of this stage to host invasion and disease progression, it is essential that this gap in knowledge be closed.^16^

The mechanisms in which *Fasciola spp.* parasites interface with their hosts has been an area of intense scientific scrutiny in recent decades, particularly focussing on excretory-secretory (ES) proteins, extracellular vesicles (EVs) and the tegument.^17–23^ The tegument has previously been investigated in *F. hepatica* given its proposed involvement in immunological processes of early infection and in-host resolution *via* antibody-dependent cellular cytotoxicity.^18^ Components of the tegument have also demonstrated antigenic properties and therefore represent an attractive target for vaccine research.^24^

Yet to be explored for *F. gigantica* are the CD59-like proteins of the Ly6 family, named for their homology to human CD59 (UniProt: P13987), a complement cascade inhibitor which regulates membrane attack complex (MAC) mediated cytotoxicity of the human complement cascade.^25^ Homologous proteins have been identified on the teguments of a range of parasitic helminths, including *Schistosoma mansoni*, *Fasciola hepatica* and *Opisthorchis viverrini*.^26–28^ These proteins exhibit conserved structures: uPAR-like domains, ten cysteines in conserved positions and a three-fingered tertiary structure which approximates to that of human CD59.^28,29^ Despite the similarity of these sequences to human CD59, it is unknown whether their functions within helminths are comparable, namely proposed dysregulation of host complement *via* interruption of MAC formation as a form of protective immunomodulation, or entirely unique.^26^ Considering that preliminary immunization trials using *S. mansoni* Ly6 family proteins demonstrate promising effects on host antibodies, complete characterisation of these proteins within *F. gigantica* has the potential to reveal a novel vaccine candidate for future research.^29–31^

To date, characterisation of the Ly6 proteins in *Fasciola spp.* has been limited to a single study in which three Ly6 proteins, FhCD59-1 to 3, with FhCD59-1 represented by seven isoforms, were identified.^27^ To the authors’ knowledge, no attempts have been made to characterise the Ly6 proteins of *F. gigantica.* Alongside the recent genome, transcriptomic data from a newly-excysted juvenile (NEJ) described herein represents a unique opportunity to investigate Ly6 proteins, given that NEJs are strongly associated with pathogenesis, and thus represent the ideal phase for intervention *via* vaccination.^32^ This study aimed to exploit the recent expansion of *F. gigantica* nucleotide databases to deliver the most comprehensive characterisation of *Fasciola* Ly6 proteins to date.

## Materials and methods

### Excystment of *Fasciola gigantica* metacercariae

*F. gigantica* metacercariae were obtained from naturally infected wild snails collected in Aligarh, Uttar Pradesh, India, by researchers at Aligarh Muslim University. Excystment was performed as reported by McVeigh *et al.*^33^ Briefly, the outer cyst walls were removed by incubation in a solution of 1% w/v pepsin, 4 mM HCl, for 90 min at 37 °C. Post pepsin incubation, cysts were washed several times in distilled water. Excystment was initiated by incubation of the cysts in 0.6% w/v sodium bicarbonate, 0.45% w/v sodium chloride, 0.4% w/v sodium tauroglycocholate, 0.025 M HCl and 0.4% w/v L-Cysteine, for up to 4 h at 37 °C. After approximately 75 min, at 10–15 min intervals, NEJs were transferred to DMEM (Life Technologies), in which they were maintained at 37 °C overnight for which excystment was completed within 18 h. NEJs were centrifuged at 400*×g* for 1 min and the DMEM removed. NEJs were washed in warmed DMEM, centrifuged at 400*×g* for 1 min and the DMEM removed. *F. gigantica* NEJs were then flash frozen in liquid nitrogen and stored at −80 °C for RNA extraction.

### RNA extraction and normalised cDNA production

In total 30 mg of *F. gigantica* NEJs were used for total RNA isolation. Total RNA (tRNA) was purified using the Qiagen RNeasy kit as per the manufacturer's instructions. NEJs were homogenised using Eppendorf micropestles and supported by passing the homogenate through a 21 gauge (0.8 μm) needle. tRNA was quantified using NanoDrop (ThermoFisher, UK). 4.5 μg of tRNA was normalised to prevent over representation of abundant transcripts and used for complimentary DNA synthesis following the Evrogen (Russia) CS010-1C protocol using the SMART approach.^34^ cDNA library production, GsuI digestion and 454 sequencing was performed at the Centre for Genomic Research at the University of Liverpool (UK) using one plate of the 454 GS FLX platform. All 454 sequence data representing the *F. gigantica* NEJ transcriptome is available through NCBI Transcriptome Shotgun Assembly (TSA) under the accession number GJHP01000000.

### Transcriptome bioinformatics

Assembly statistics were generated using the ‘assembly-stats’ JavaScript repository.^35^ Mapping of the NEJ transcriptome against the publically available *F. gigantica* genome (GenBank accession GCA_006461475.1) was performed using EasyBuild BLAST+ (version 2.11.0) with an E-value cut-off of 1 × 10^−10^ and a minimum bit score of 70.^8^ Gene ontology (GO) term and InterPro annotations were then produced for the complete *F. gigantica* NEJ transcriptome using the Functional Annotation workflow within OmicsBox.^36^

### Gene ontology (GO) term enrichment of the *Fasciola gigantica* newly-excysted juvenile transcriptome against the genome

GO annotations for the draft genome predicted protein data (GenBank GCA_006461475.1) were generated using PANNZER2 for comparison with GO outputs previously generated for the transcriptome.^8,37^ Gene identifiers were mapped between the two datasets using reciprocal BLASTs with an *E*-value threshold of 0.1 using in-house scripts. GO term enrichment analysis was then performed using GOAtools (version 0.5.9) script in Python using paired *t*-tests and FDR corrected *P*-values.^38^ Child GO terms were not propagated to parents as an attempt to reduce inheritance over-representation.^39^ Significant enrichment was measured at two levels: significant, *q* < 0.05 and highly significant, *q* < 0.001.

### Bioinformatic characterisation of *Fasciola gigantica* and *Fasciola hepatica* Ly6s

Sequences for Ly6 members identified previously by Shi *et al.* and Chalmers *et al.* were retrieved for *F. hepatica* and *S. mansoni* members, respectively.^27,28^ Recently identified SmLy6 members Smp_202630 and Smp_064430 were also included (putatively identified by Chalmers *et al.*).^28^ Accession numbers for the *S. mansoni* proteins were updated from Chalmers *et al.* to the latest versions using WormBase ParaSite (version WBPS14) and the *S. mansoni* Meta RNAseq library (V7).^27^

tBLASTn searches were performed using the reference sequences against the *F. gigantica* newly excysted juvenile (NEJ) transcriptome, using the local database function within BioEdit (version 7.2.5.).^40^ Sequences with *E*-values smaller than the designated 1 × 10^−5^ cut-off were subsequently retrieved and translated into proteins using ExPASy translate.^41^ BLASTp searches of an adult *F. gigantica* predicted protein library and draft genome were subsequently performed using the same reference sequences, in addition to putatively identified Ly6 proteins from the NEJ transcriptome, using the same *E*-value cut-off.^8,12^

Ly6 proteins of *F. hepatica* were subsequently characterised in the same manner as those found in *F. gigantica*, with the exception that the previously characterised *F. gigantica* orthologues were also incorporated into the query sequence list during the BLAST searches of the transcriptomes. Complete life-stage specific characterisation was performed using a *F. hepatica* adult predicted protein library, as well as six stage-specific assemblies (egg; metacercaria; newly excysted juveniles at 1, 3 and 24 hours; adult) provided in curated format by Queens University, Belfast.^7,42^ Characterised Ly6 proteins from *F. hepatica* were also identified in the Liverpool *F. hepatica* genome (accession PRJEB25283) in WormBase BLASTp using standard parameters and an *E*-value cut-off of 1 × 10^−5^. Interspecies, *F. gigantica* to *F. hepatica*, orthologues were defined as >90% identity over 100 continuous residues.

Sequences were screened for uPAR-domain features; namely the presence of at least ten cysteine residues within 120 amino acids, a C^1^–XX–C^2^ N-terminal motif (where X represents any amino acid) and a C^10^–N motif within the C-terminal (Pfam accession PF00021). Additional canonical Ly6 protein features were also identified in accordance with the literature.^27,28^ This included a signal peptide identified with Signal-P 5.0 and a GPI-anchor identified using the big-PI GPI modification site predictor.^44,45^

*De novo* tertiary structural predictions were generated for the domain region of the mature proteins (C-terminal pre-C^1^XXC^2^ motif and N-terminal post-GPI anchor removed) as previously described.^28^ Briefly, structural modelling was done using Rosetta following a *de novo* protocol generating 1000 full-atom models.^46,47^ Structural decoys were clustered and top scoring models as per Rosetta scores were visualised and analysed using PyMol (version 2.3.3) (Schrödinger, LLC).

### Phylogenetics analysis

Phylogenetic analysis using domain region protein sequences from the characterised Ly6 sequences from *F. gigantica*, *F. hepatica* and *S. mansoni* was performed using MEGAX (version 10.0.5).^48^ To aid in the isolation of fasciolid-specific clades, proteins with Ly6 domain features from *Clonorchis sinensis* (PRJDA7281), *Opisthorchis felineus* (PRJNA413383) and *Opisthorchis viverrini* (PRJNA222628) were also added *via* BLASTp FgLy6 homology at 1 × 10^−5^. Maximum likelihood reconstruction was performed with 2000 bootstraps, JTT model substitution and otherwise default parameters. The resulting distance-scaled tree was separated into clades according to a minimum ancestral root support value of 40. Life stage expression data, extrapolated from sequence presence or absence in each of the transcriptomic and genomic databases searched, was also added.

### Proteomic annotation

Querying of available *F. hepatica* proteomic databases was performed to retrieve representative EV and tegument expression data for each of the newly identified proteins.^18,22^ Expression data for the *S. mansoni* orthologues was also retrieved for comparison between orthologues.^28^

## Results

### *Fasciola gigantica* newly excysted juvenile transcriptomic profile

Assembly statistics for the *F. gigantica* NEJ transcriptome are summarised in Table 1. The NEJ transcriptome contained a total of 16 551 transcripts, of which 4,031 could not be classified by BLASTx, InterPro, or GO mapping/annotation; 2097 produced BLAST/InterPro hits only; 960 produced were successfully mapped and 9463 reached completion with full GO annotation. A direct count of GO terms identified the top five annotation terms for each class to be as follows: Biological Process (BP) - protein phosphorylation, oxidation–reduction processes, translation, proteolysis and regulation of transcription (DNA-templated); Cellular Component (CC) – integral component of membrane, nucleus, membrane, cytoplasm and ribosome; and Molecular Function (MF) – ATP binding, metal ion binding, protein binding, nucleic acid binding and RNA binding. When mapped against the available *F. gigantica* genome, 97% of the 16551 assembled transcripts produced strong hits, with 495 additional transcripts revealed in the NEJ (supplementary file 1, ESI†).

**Table 1:** Key assembly statistics for the *F. gigantica* NEJ transcriptome, generated using ‘assembly-stats’ JavaScript and the OmicsBox functional analysis workflow

Metric	*F. gigantica* NEJ transcriptome.
Total Sequences (Contigs)	16 551
Mean sequence length (bp)	799
Longest transcript (bp)	4239
Contig N50 length (bp)	988
Contig N90 length (bp)	442
GC content (%)	45.14
AT content (%)	54.86
*N* content (Gapped) (%)	0.00

### Gene ontology (GO) term enrichment

To determine any functional representation in the *F. gigantica* NEJ transcriptome compared to the newly produced draft genome a GO-term enrichment analysis was performed. A total of 376 GO-terms were compared across the datasets (supplementary file 2), with biological processes (BP) most heavily represented (*n* = 203), followed by cellular components (CC, *n* = 89) and molecular function (MF, *n* = 84). 13 terms were found to be significantly enriched in the NEJ (*p* < 0.05) with 7 terms reaching significance at *p* < 0.001. Enrichment was observed mostly in CC terms (*n* = 9), followed equally by BP (*n* = 2) and MF (*n* = 2). The most significantly enriched terms (*p* < 0.001) included translation (GO:0006412), mRNA splicing *via* spliceosome (GO:0000398), ribosome (GO:0005840), mitochondrion (GO:0005739), mitochondrial matrix (GO:0005759), RNA binding (GO:0003723) and structural component of ribosome (GO:0003735).

Conversely, 24 terms were found to be significantly enriched in the genome (*p* < 0.05), with 14 of these reaching significance at *p* < 0.001. Genome enriched terms were more evenly distributed between BP, CC and MF, with 9, 5 and 10 terms respectively, and included potassium ion transmembrane transport (GO:0071805), multicellular organismal development (GO:0007275), endopeptidase activity (GO:0004175), cysteine-type peptidase activity (GO:0008234), dynein intermediate light chain binding (GO:0051959) and structural constituent of cytoskeleton (GO:0005200).

### Protein domain analysis by transcript frequency

Transcript domain analysis was performed on the *F. gigantica* NEJ transcriptome to determine the presence of commonly targeted domains in helminth development research. Domains were ranked according to frequency (measured in number of annotated transcripts) and the top 20 domains were selected. A total of 1,563 domains with unique InterPro handles were identified, with a sequence frequency ranging from 1 to 109 across all identified domains. Several domains of relevance to vaccine research were included in the top 20 (Table 2).

**Table 2:** Top 20 identified InterPro domains in the NEJ *F. gigantica* transcriptome according to number of sequences per domain. Example helminth vaccine or drug candidates for each domain type given for reference, where possible. Full domain annotations are provided in the supplementary files

InterPro ID (IPR-).	Domain.	Number of NEJ transcripts.	Example vaccines or drug candidates
002048	EF-hand	109	Tegument-allergen like (TAL) proteins.^49^ Calpains^50^
000504	RNA recognition motif	102	—
000719	Protein kinase	98	ePKs (conventional protein kinases)^51^
017986	WD40-repeat-containing	73	—
005225	Small GTP-binding protein	63	—
001841	Zinc finger, RING-type	45	—
013087	Zinc finger, C_2_H_2_-type	44	—
020683	Ankyrin repeat-containing	36	—
013026	Tetratricopeptide repeat-containing	33	—
001650	Helicase, C terminal	32	—
008139	Saposin B type	31	Saposins.^52^
000477	Reverse transcriptase	30	—
000608	Ubiquitin-conjugating enzyme E2	29	—
001452	SH_3_	29	—
014001	Helicase superfamily 1/2, ATP binding	29	—
017452	GPCR, rhodopsin-like, 7TM	28	G-coupled protein receptors^53^
001781	Zinc finger, LIM-type	28	—
001623	DnaJ	28	—
004088	K homology, type 1	27	—
001478	PDZ	26	Syntenin^54^

### Characterisation of Ly6 proteins of *Fasciola gigantica*

**Fig. 1:**
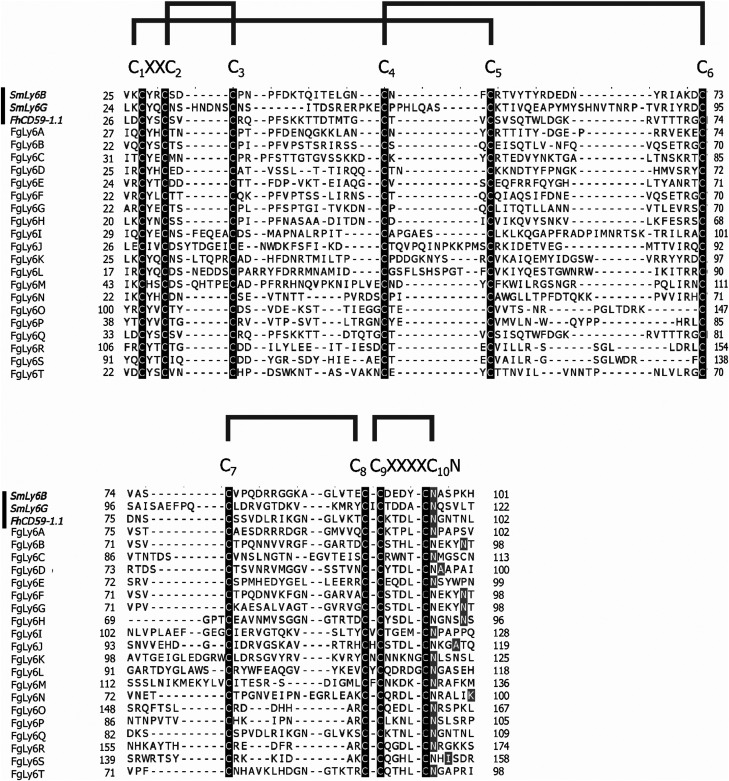
Multiple sequence alignment of domain region protein sequences (C_1_XXC_2_ motif to GPI anchor, N-terminal signal peptide and C terminal hydrophobic region removed) for the twenty novel FgCD59 members. Reference sequences from *S. mansoni* (SmLy6B and SmLy6G) and *F. hepatica* (FhCD59-1.1) are included as denoted by the black bar to the left of the alignment. Conserved motifs are indicated above the alignment, with the ten cysteines highlighted by the black boxes. GPI anchors are denoted in grey. Predicted cysteine bonds are indicated by the grey brackets above the alignment. Alignments produced in CLUSTAL Omega and annotated in Jalview.

**Fig. 2:**
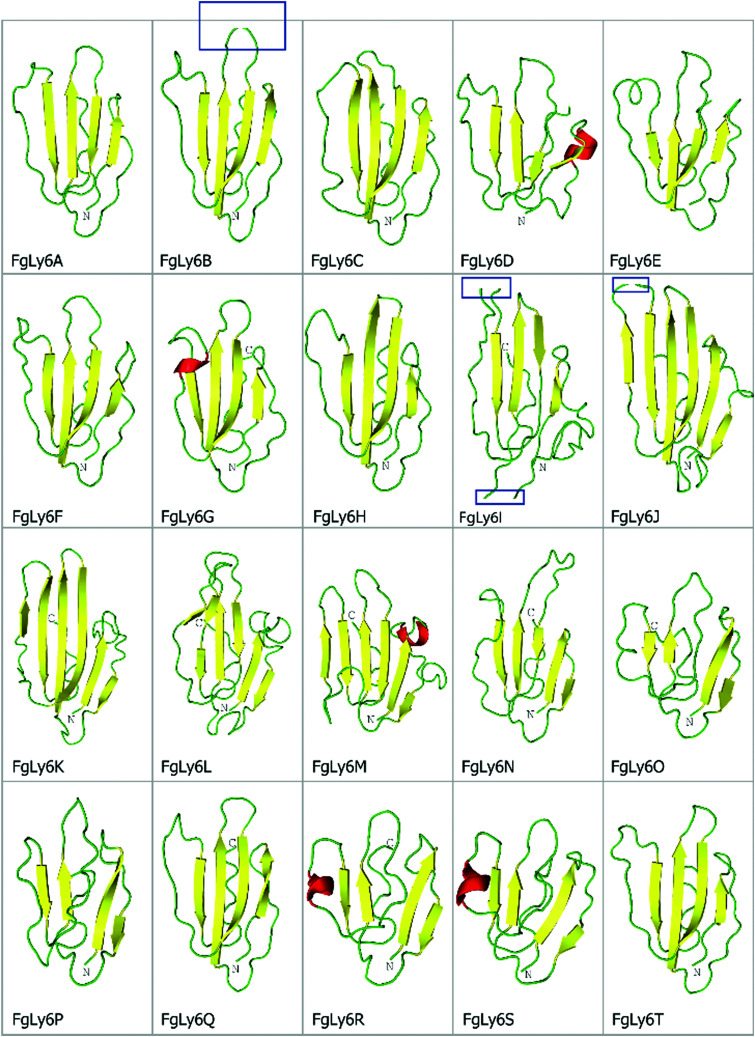
*De novo* tertiary structural predictions for the twenty novel FgLy6s, produced using Rosetta. Beta strands, loops and helices are represented in yellow, green and red, respectively. Note the conserved central fold and presence of a defined ‘three finger’ motif, primarily characterised by three protruding loops at the top of the protein structure. C and N terminals are also annotated, where visible. The strand breaks visible in FgLy6-B, -I and -J (blue boxes) are artefacts of uniform snapshot scaling and are not present in the full model.

As Ly6 proteins are unlikely to be identified during a domain search, additional homology BLAST (tBLASTn or BLASTp) searches were performed using known Ly6 proteins from *F. hepatica* and *S. mansoni* against the NEJ transcriptome, the adult *F. gigantica* transcriptome and an *F. gigantica* genome.^8^ Twenty hit sequences displayed uPAR-domain features in approximately conserved locations when aligned with *F. hepatica* and *S. mansoni* Ly6 reference sequences (Fig. 1). These sequences were arbitrarily designated as FgLy6-A through T (Table 3). Of these, five (FgLy6-C, -E, -F, -G and -H) were only present in the NEJ. Three additional FgLy6s, -Q, -R and -S, were found in the adult and genome datasets only, with no NEJ equivalent at 1 × 10^−5^. Six additional sequences met the 1 × 10^−5^ threshold but contained no domain features and were removed from further analysis. Following uPAR-domain characterisation, the putative FgLy6 sequences were screened for secondary Ly6 features, including a signal peptide and a GPI anchor. Of the twenty FgLy6s, eighteen had signal peptides that could be confidently predicted by Signal P 5.0 and all twenty sequences had GPI-anchors present in their C-terminals. All predictions also fell in approximately conserved locations within the FgLy6 sequences when aligned with *F. hepatica* and *S. mansoni* reference sequences (Fig. 1). Finally, tertiary structural predictions for the twenty putatively identified FgLy6s were produced using *de novo* modelling. All twenty FgLy6s converged into the conserved three-finger domain fold and demonstrate highly conserved core structures, as shown in Fig. 2.

**Table 3:** Summary of all FgLy6s identified by BLAST similarity to known *F. hepatica* and *S. mansoni* sequences (at *E* < 1 × 10^−5^) and presence of uPAR-domain features. FgLy6s were identified across three databases (NEJ transcriptome, adult transcriptome and *F. gigantica* genome). Signal peptides were predicted using Signal P 5.0. Transmembrane domains were predicted using TmPred. Protein sequence data corresponding with the NEJ transcript IDs are also available in Supplementary Data

Ly6	GenBank accession	Sequence length (amino acids)	Signal peptide	C-terminal transmembrane domain	NEJ *F. gigantica* transcript ID.	Transcriptomic support in adult *F. gigantica.*	*F. gigantica* genomic support.	*F. hepatica* orthologue identified. *Δ*
A	TPP67930	127	+	+	01777	+	+	+^^b^^
B	TPP57504	122	+	+	05428	+	+	−
C	TPP61687	132	+	+	04026	−	+	+^^b^^
D	TPP58533	121	+	+	11947	+	+	+^^ab^^
E	—	109	+	−	11274	−	−	−*
F	TPP58767	122	+	+	05429	−	+	+^^b^^
G	TPP66235	122	+	+	11065	−	+	+^^b^^
H	TPP66236	120	+	+	14360	−	+	+^^b^^
I	TPP67438	150	+	+	10885	+	+	+^^ab^^
J	TPP56171	143	+	+	10181	+	+	+^^ab^^
K	TPP58682	152	+	+	11200	+	+	+^^ab^^
L	TPP59839	138	+	+	01142	+	+	+^^ab^^
M	TPP61345	153	+	+	09765	+	+	+^^ab^^
N	—	117	+	+	02813	+	+	+^^ab^^
O	TPP67399	189	+	+	06228	+	+	+^^ab^^
P	TPP57978	127	−	+	02241	+	+	−
Q	TPP67483	129	+	+	−	+	+	+^^b^^
R	TPP67398	197	+	+	−	+	+	−*
S	TPP57976	166	−	−	−	+	+	+^a^
T	TPP67484	121	+	+	−	−	+	−*

### Expansion of the Ly6 protein family of *Fasciola hepatica*

Following the complete *in silico* characterisation of the FgLy6s leading to a considerable expansion on known *Fasciola* species Ly6 members, a follow up analysis was performed to determine if orthologous *F. hepatica* proteins could be identified. Orthologous proteins were defined as those with > 90% identity over at least 100 continuous amino acids. Of the twenty FgLy6s, fifteen had *F. hepatica* orthologues in at least one transcriptomic dataset (Table **3**). FgLy6-E, -R and -T also matched to *F. hepatica* orthologous sequences on direct alignment but were marginally below the cut-off threshold during a BLAST search (85% ID over 109 residues, 90% ID over 81 residues and 86% ID over 192 residues for E, R and T respectively). FhLy6-A and FhLy6-Q represented previously characterised *F. hepatica* Ly6 proteins (FhLy6-2 and FhLy6-1.1 respectively).^27^

In addition to the sixteen FgLy6-orthologous proteins, three additional proteins exclusive to *F. hepatica* were also identified (FhLy6 -U, -V and -W) using transcriptomic data. Of these, one had already been identified in previous works (FhCD59-3, given here as FhLy6-V) and was the only *F. hepatica* specific protein with genomic support.^27^ The remaining two had not previously been identified.

### Phylogenetic analysis

**Fig. 3:**
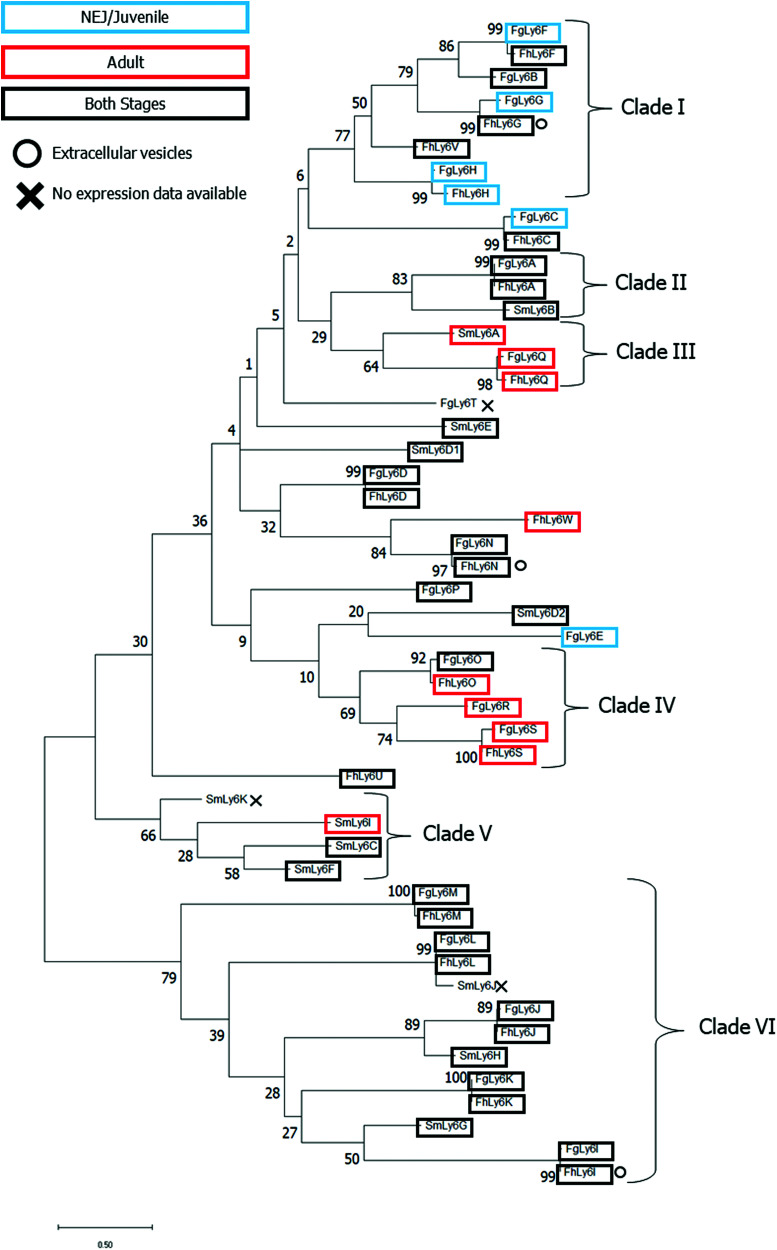
Phylogenetic analysis of all known CD59 family members of *F. gigantica*, *F. hepatica* and *S. mansoni*. Sequences were processed to remove the N-terminal sequence prior to the C_1_XXC_2_ motif and C-terminal post GPI anchor before alignment using Clustal Omega. SmLy6D, a double-domain CD59, was further divided into two (D1 and D2). Maximum likelihood analysis was performed in MegaX to 2000 bootstraps with JTT substitution. Life stage-specific expression profiles are indicated by boxes in addition to *in vitro* expression data where applicable. Clades were defined with an ancestral support cut-off of ≥ 40.

A maximum-likelihood phylogenetics analysis with 2000 bootstraps was performed on domain-region Ly6 proteins from *F. gigantica*, *F. hepatica* and *S. mansoni*. Existing *F. hepatica* sequences were renamed to correspond with the *F. gigantica* alphabetic nomenclature. The phylogenetic relationships between all three species’ Ly6 proteins and associated transcriptomic and proteomic expression data are provided in Fig. 3.

Several clades, defined by an ancestral root support value ≥40%, were resolved, including two clades which were unique to the two *Fasciola* spp. (clades I and IV, clade root support value of 77% and 69%, respectively). Clade I was also the only defined clade to contain juvenile-specific FgLy6s (F, G and H). Six FgLy6s could not be grouped into a clade and appeared to be relatively distinct from both *S. mansoni* proteins and each other (FgLy6-C, -D, -E, -N, -P and -T), with the only closely related proteins being their FhLy6 orthologues, when present. Repeated phylogenetic analysis to include putatively identified *Opisthorchis spp.* and *C. sinensis* Ly6 proteins revealed that FgLy6-B, -F, -G, -H and -V remain from *S. mansoni* when contextualised by orthologues from other trematodes (root support value 62%, supplementary file 4, ESI†).

Querying of published proteomic databases for *F. hepatica* was also performed to attempt to localise the Ly6 members within the parasite. FhLy6s which matched to accessions in the Davis *et al.* extracellular vesicle proteome were distributed throughout the phylogenetic tree, with three FhLy6s, -G, - I and -N, found to be expressed in at least one experimental replicate.^22^ All three were found to be expressed in both life stages, with two of the three belonging to distinct clades (I and VI for G and I, respectively). Querying of the Wilson *et al.* tegumental transcriptome returned no hits for any of the FhLy6 members.^18^

## Discussion

*F. gigantica*, despite being a zoonotic trematode of considerable global socio-economic importance, has thus far been largely neglected in respect to molecular characterisation, especially in comparison to its temperate relative, *F. hepatica*.^12^ The 18 h NEJ transcriptome described here represents the first to be produced for this ontogenetic stage of *F. gigantica* and provides an exciting opportunity to explore the stage-specific expression of key target proteins. To date, novel proteins have already been revealed through the analysis of this important life stage transcriptome including a Zeta class glutathione transferase (GST) and a novel second Sigma class GST isoform.^55^ Interestingly, several of the domains highlighted within the top twenty analysis indicate that further expansion of potential vaccine-candidates may also be possible.

For example, the top domain hit, EF-hand domain proteins, include the tegument-allergen-like (TAL) proteins, which have previously been identified as potential vaccine candidates due to their association with host immune responses, specifically IgE-based.^49^ In *S. mansoni*, thirteen TAL proteins have been identified and characterised in total.^49,56^ Though TAL-family proteins have also been studied in both *F. gigantica* (FgCaBPs) and *F. hepatica* (FhCaBPs), far fewer are known at present (four in *F. gigantica*, three in *F. hepatica*). Also represented among the EF-hand domain proteins are the calpains, a family of cysteine proteases which have been highlighted as potential vaccine candidates in schistosomes.^50,57^ Two calpains of *F. hepatica* have previously been identified using ‘omics data, while *F. gigantica* orthologues remain formally unidentified.^18^ While EF-hand domains are not exclusive to these two families, putative annotation of the 109 EF-hand transcripts revealed the presence of 13 tegument antigens and 5 calpains, indicating the potential for family expansion and isoform discovery in *F. gigantica* using this transcriptome.^58,59^

In addition to the presence of several domains of interest, GO term enrichment analysis indicated several significantly enriched terms in the 18 h NEJ transcriptome compared to genomic data. The results of this study were comparable to previous work by Zhang *et al.* (2019), wherein GO enrichment analysis was performed between various *F. gigantica* life stages, including juvenile vs adult.^13^ Though there were no overlapping GO terms between the two studies, this is likely an artefact of the difference in term propagation depth between analyses, as well as the age of the juvenile studied, 18 h NEJ *vs.* 70 days post infection juveniles. Several common ‘themes’ were observed in the descriptive terms, including signalling-, microtubule- and cytoskeletal-associated terms. This likely reflects the considerable development required to transition into definitive host from the environmental stages, as well as responses to requirements for immune-interfacing and migration.^13,60,61^

During this study, the Ly6 family proteins were targeted for further analysis, as it was unlikely that they would be picked up by a domain-level query. This proved to be the case in the NEJ transcriptome, as only a single uPAR-like domain protein was annotated during the domain analysis; a contrast to the twenty subsequently characterised. Further investigation also noted that this sequence did not have all the features required to be classified as a uPAR-domain protein (at least ten cysteine residues, C1–XX–C2 N-terminal motif, C10–N motif; isotig 18073, annotated with InterPro accession IPR016054) and was not a partial sequence, therefore was classified as a mis-annotation. This demonstrates the need for alternative discovery pipelines for Ly6 family proteins aside from domain identification.

The considerable expansion of the Ly6 protein family described herein is extremely positive for the progression of vaccine research in *Fasciola spp*. trematodes. Characterisation of the *S. mansoni* Ly6 proteins revealed several candidates able to stimulate a protective immune response, notably SmLy6D.^28^ Preliminary vaccine trials using SmLy6D demonstrated a reduction in adult worm burden of >50% in a mouse model, with significant reductions in egg production and clinical signs of inflammation also observed.^30^ Though no direct homologue to SmLy6D was found in this study, the high level of diversity observed in the *Fasciola spp*. Ly6 proteins is encouraging for the discovery of an equivalent candidate. Additionally, the high level of orthology demonstrated between *F. gigantica* and *F. hepatica* Ly6 proteins (15 of 20 FgLy6s with orthologues present in *F. hepatica* at > 90% sequence identity over 100 amino acids) is also encouraging for the cross-species coverage of any vaccine candidates progressed from this research.

On complete characterisation of the *Fasciola* spp. Ly6 proteins, phylogenetic analysis was performed on all sequences, in addition to the *S. mansoni* homologues, to determine if the sequences could be clustered into distinct Ly6 groups. Results demonstrated the presence of several distinct clades, with the previously defined life-stage expression patterns from the FhLy6s reflected by their newly characterised FgLy6 orthologues.^7,43^ This support of stage-specific expression by distinct phylogenetic clades has previously been observed in other vaccine candidate families, such as the cathepsin L proteases.^10,62,63^ Ly6 proteins S, Q and H were found to be expressed only in the adult (S and Q) or juvenile stages (H) across both fasciolids, which indicates the potential for stage-targeted intervention strategies. FgLy6-F, -G and -C were also noted to be juvenile specific, however, this juvenile specific cluster conflicted with their FhLy6 orthologues, which were expressed in both stages. This is likely an artifact of improved transcriptomic quality in the newer NEJ transcriptome in comparison to the older adult *F. gigantica* transcriptome.^12^ Therefore, these isotypes could also be expressed in both stages in *F. gigantica*. Considering the extent to which the *S. mansoni* Ly6s have been studied and the expansive available ‘omic data in this parasite, it is unlikely that there are undiscovered SmLy6s which would conform to the *Fasciola spp*. distinct clades and thus it is likely that these eight family members, B, F, G, H, O, R, S and V, are distinct within the platyhelminthes.^28,64^ Notably, no orthologues of the primary *S. mansoni* vaccine candidate, SmLy6D were identified in either of the *Fasciola spp*.

Although *S. mansoni* Ly6s B, D and F have previously been identified as major antigens of the tegument, little is known about the functions of tegumental Ly6 proteins.^28^ FhLy6 proteins have been found not to contain essential active sites to fulfil roles as complement inhibitors, however the presence of a GTPase may indicate that Ly6 proteins are capable of signalling.^18,65^ None of the newly identified FhLy6s appeared to be present in the tegumental proteome. However, additional proteomic data with increased proteomic sensitivity is required to confirm this, as Ly6 retrieval appears to be highly dependent on the techniques observed.^27,66^ Two of the *S. mansoni* Ly6 proteins which have previously been identified in the tegument have closely related *Fasciola* spp. homologues (Q with SmLy6A, support value 61, and A with SmLy6B, support value 81, respectively) and may therefore be expected to be expressed in the tegument.^28^

Whilst additional transcriptomic and proteomic resources are now available for both *F. gigantica* and *F. hepatica*, published data is frequently orphaned from raw sequence files and instead rely on indexing using historic BN-numbers, which refer to an outdated, previous version of the PRJEB25283 *F. hepatica* genome.^13,22,23,67^ In light of the newly published *F. gigantica* genomes, and the transcriptome described herein, reflection upon existing resources to incorporate these updates would be highly valuable, as the current form of indexing precludes any further analysis. Thus, there is a real opportunity to reanalyse historic datasets, such as the study by Zhang *et al.*, to support future analyses on key target protein families.^13^

## Conclusions

The considerable expansion of the Ly6 protein family described in this study demonstrates the value of novel transcriptomic resources, especially in the invasive stages. Whilst additional confirmatory research (for example subcloning and RNAi) would ideally need to be performed, leveraging ‘omics resources in parasite research represents a relatively low-cost method for the identification of novel anthelmintic targets, as well as allowing the characterisation of a range of key biological and molecular functions. Preliminary exploration of the NEJ transcriptome described herein indicates there are still many protein families of interest that could be explored and thus this transcriptome represents an attractive resource for future therapy and diagnostic research.

## Data availability

The 18 h NEJ *F. gigantica* Transcriptome Shotgun Assembly project has been deposited at DDBJ/EMBL/GenBank under the accession GJHP00000000. The version described in this paper is the first version, GJHP01000000. In addition, The NEJ *F. gigantica* transcriptome is available to interrogate *via*https://sequenceserver.ibers.aber.ac.uk/.

## Author contributions

Conceptualization – P. M. B., A. G. M., I. W. C. and R. M. M.; data curation – S. D. D.; formal analysis – S. D. D., N. F. F., R. M. M., M. S. and D. S.; funding acquisition – P. M. B., A. G. M., S. M. A. A., K. S., M. R., G. R., P. M., R. O. M. and N. F. F.; investigation – S. M. A. A., K. S., M. R. and G. R.; methodology – R. M. M., I. W. C., N. F. F. and M. S.; supervision – R. M. M., P. M. B., A. G. M. and I. W. C.; writing – original draft – S. D. D., R. M. M. and I. W. C.; writing – review & editing – S. D. D., R. M. M., I. W. C., N. F. F., M. S. and P. M. B.

## Conflicts of interest

The authors declare there are no conflicts of interest.

## Supplementary Material

MO-018-D1MO00254F-s001

MO-018-D1MO00254F-s002

MO-018-D1MO00254F-s003

MO-018-D1MO00254F-s004

MO-018-D1MO00254F-s005

MO-018-D1MO00254F-s006
