# Supplementary figures and images for: In silico characterisation of the complete Ly6 protein family in Fasciola gigantica supported through transcriptomics of the newly-excysted juveniles
Source: Mol Omics. 2021 Nov 8;18(1):45–56. doi: 10.1039/d1mo00254f (PMC8763315; doi:10.1039/d1mo00254f)

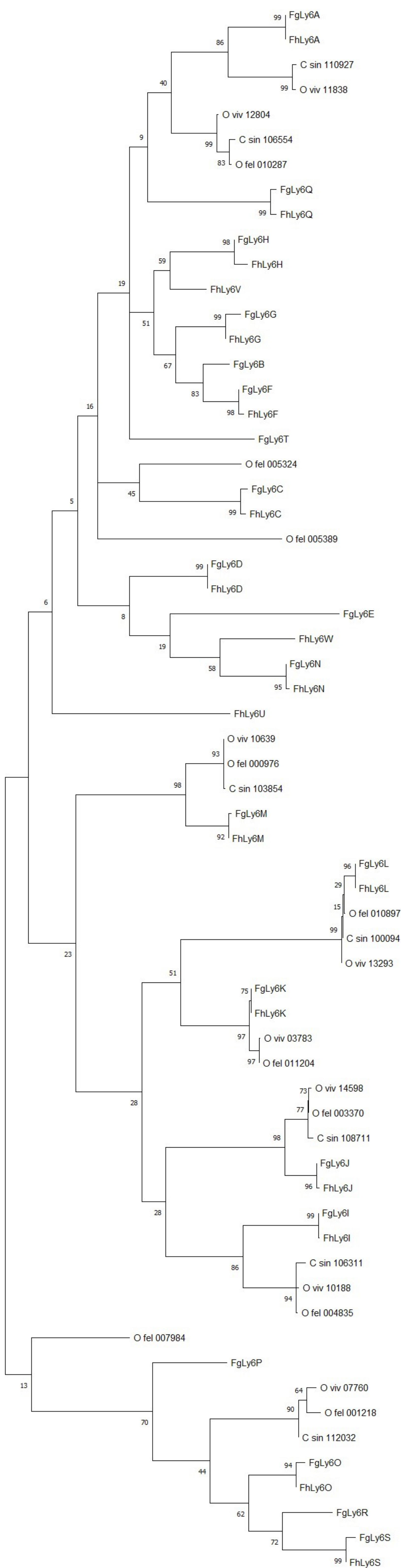

0.50

Supplement: MO-018-D1MO00254F-s004 [file MO-018-D1MO00254F-s004.pdf]
